# ABO blood group system and the coronary artery disease: an updated systematic review and meta-analysis

**DOI:** 10.1038/srep23250

**Published:** 2016-03-18

**Authors:** Zhuo Chen, Sheng-Hua Yang, Hao Xu, Jian-Jun Li

**Affiliations:** 1 Division of Dyslipidemia, State Key Laboratory of Cardiovascular Disease, Fu Wai Hospital, National Center for Cardiovascular Diseases, Chinese Academy of Medical Sciences, Peking Union Medical College, BeiLiShi Road 167, Beijing 100037, China; 2 Graduate School, Beijing University of Chinese Medicine, BeiSanHuan East Road 11, Beijing 100029, China; 3 Cardiovascular Diseases Center, Xiyuan Hospital, China Academy of Chinese Medical Sciences, XiYuanCaoChang 1, Beijing 100091, China

## Abstract

ABO blood group system, a well-known genetic risk factor, has clinically been demonstrated to be linked with thrombotic vascular diseases. However, the relationship between ABO blood group and coronary artery disease (CAD) is still controversial. We here performed an updated meta-analysis of the related studies and tried to elucidate the potential role of ABO blood group as a risk factor for CAD. All detectable case-control and cohort studies comparing the risk of CAD in different ABO blood groups were collected for this analysis through searching PubMed, Embase, and the Cochrane Library. Ultimately, 17 studies covering 225,810 participants were included. The combined results showed that the risk of CAD was significantly higher in blood group A (OR = 1.14, 95% CI = 1.03 to 1.26, p = 0.01) and lower in blood group O (OR = 0.85, 95% CI = 0.78 to 0.94, p = 0.0008). Even when studies merely about myocardial infarction (MI) were removed, the risk of CAD was still significantly higher in blood group A (OR = 1.05, 95% CI = 1.00 to 1.10, p = 0.03) and lower in blood group O (OR = 0.89, 95% CI = 0.85 to 0.93, p < 0.00001). This updated systematic review and meta-analysis indicated that both blood group A and non-O were the risk factors of CAD.

In 1901, Karl Landsteiner, a Viennese MD and pathologist, discovered ABO blood group system which was the first human blood group^1^. From then on, studies on relation of ABO blood group system to various diseases have never been interrupted for a century, even in the popular era of gene detection, as ABO blood group is inherent in human’s body and easily to be tested.

It has been reported that ABO blood group system is associated with cognitive impairment^2^, preeclampsia^3^, bleeding, neoplastic diseases^4^, and even longevity^5^. Among all of those studies, the mechanism of relationship between ABO blood group and venous thrombosis is elucidated^6^, and its major determinants are von Willebrand factor (vWF) and coagulation factor VIII^7^ which result in thrombosis. This interesting finding makes a theoretical hypothesis that ABO blood group may also be related to risk of coronary artery disease (CAD) and myocardial infarction (MI). Unfortunately, results of previous relevant studies are currently not convincing due to inconsistent conclusions. And previous studies including original observations and meta-analysis^8^,^9^,^10^ mainly paid attention to the blood group non-O and O, ignoring the blood group A and other blood types. Moreover, in those studies, links of ABO blood group with MI was often focused on; however, the relation between risk of CAD and ABO blood group was carelessly overlooked. Therefore, this updated systematic review and meta-analysis aims to evaluate the relationship between CAD and each type of ABO blood group.

## Results

### Description of included studies

**Figure 1:**
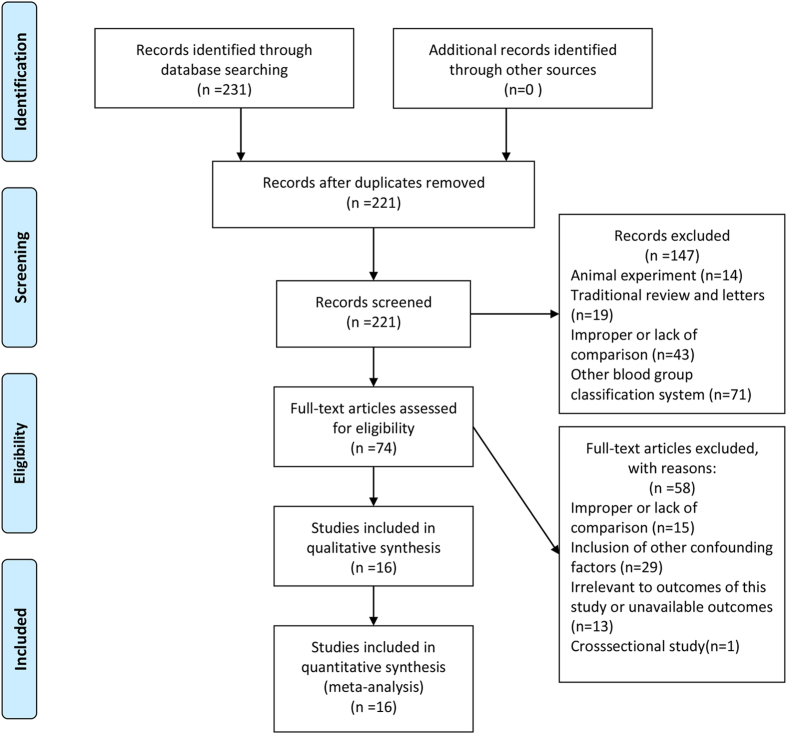
Flow-chart of study selection.

Two hundred and thirty-one studies (231 from Pubmed and 0 from the Cochrane Library) were identified from two databases. Among them, 10 records were removed on account of duplicates. By screening titles and abstracts, we excluded 147 records on account of animal experiments, traditional reviews, improper or lack of comparison, or other blood group classification systems rather than ABO blood type. By browsing full-text articles, we excluded 58 records because of improper or lack of comparison, other confounding factors, irrelevant to the outcomes of this study and unavailable outcomes. At last, a total of 16 articles^11^,^12^,^13^,^14^,^15^,^16^,^17^,^18^,^19^,^20^,^21^,^22^,^23^,^24^,^25^,^26^ which met inclusion criteria were included into this systematic review. A flow-chart of study selection was generated according to the PRISMA requirements (Fig. 1).

### Study characteristics

One^15^ of these 16 articles contained 2 studies. All the 17 studies were published in English from 1961 to 2014. Eleven articles^11^,^13^,^14^,^16^,^17^,^19^,^21^,^22^,^23^,^25^,^26^ were case-control studies, 2 articles^12^,^15^ were prospective cohort studies and 3 articles^18^,^20^,^24^ were retrospective cohort studies. Finally, a total of 225,810 patients were included. All studies described race and characteristic of the two groups. Nine studies^12^,^16^,^17^,^19^,^20^,^21^,^22^,^23^,^24^ merely mentioned MI. Two studies^12^,^17^ only differentiated blood group non-O from blood group O. The remaining studies described all blood types. (Basic characteristics of included studies were presented in Table 1 and blood types distribution and outcome definitions of included studies were presented in Table 2, and Newcastle-Ottawa Scale (NOS) table was shown in Table 3).

### Main, subgroup and sensitivity analysis

**Figure 2:**
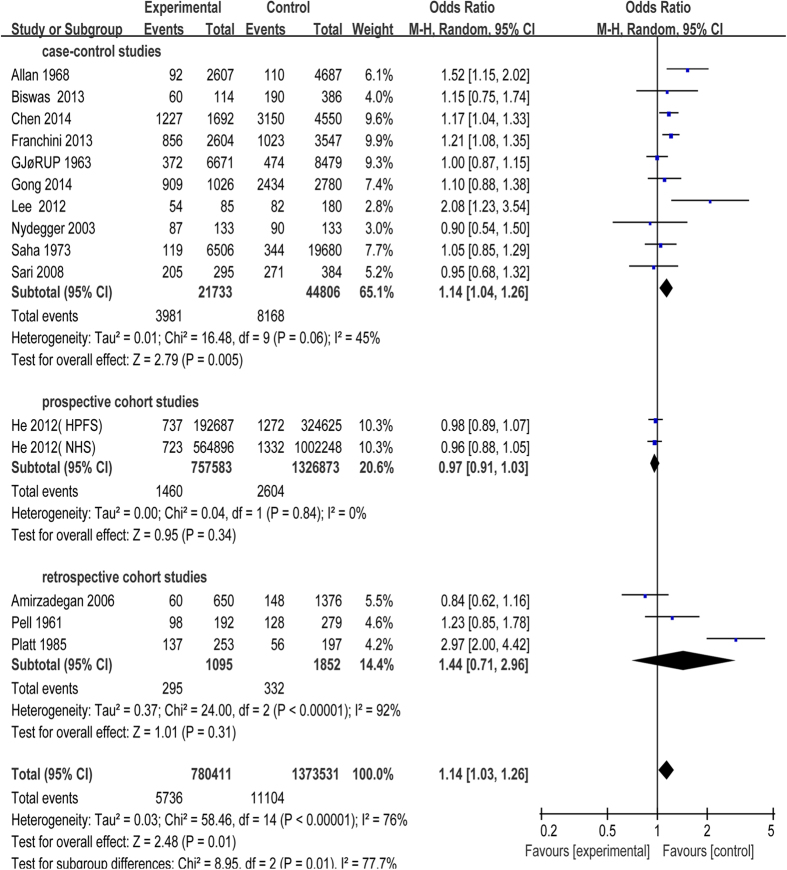
Forest plot of blood group A.

**Figure 3:**
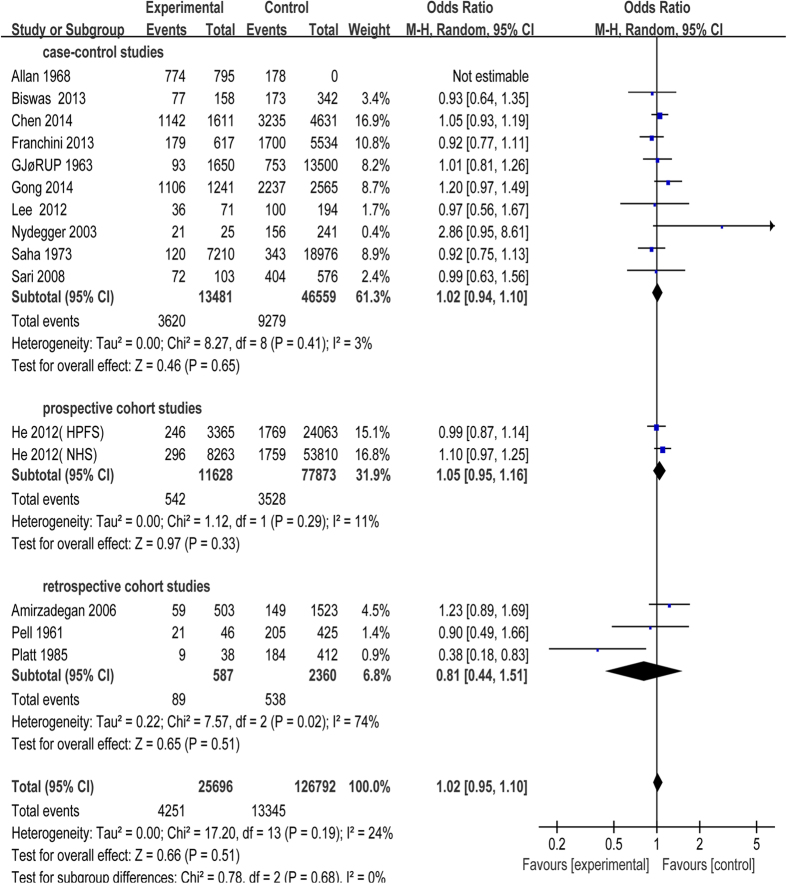
Forest plot of blood group B.

**Figure 4:**
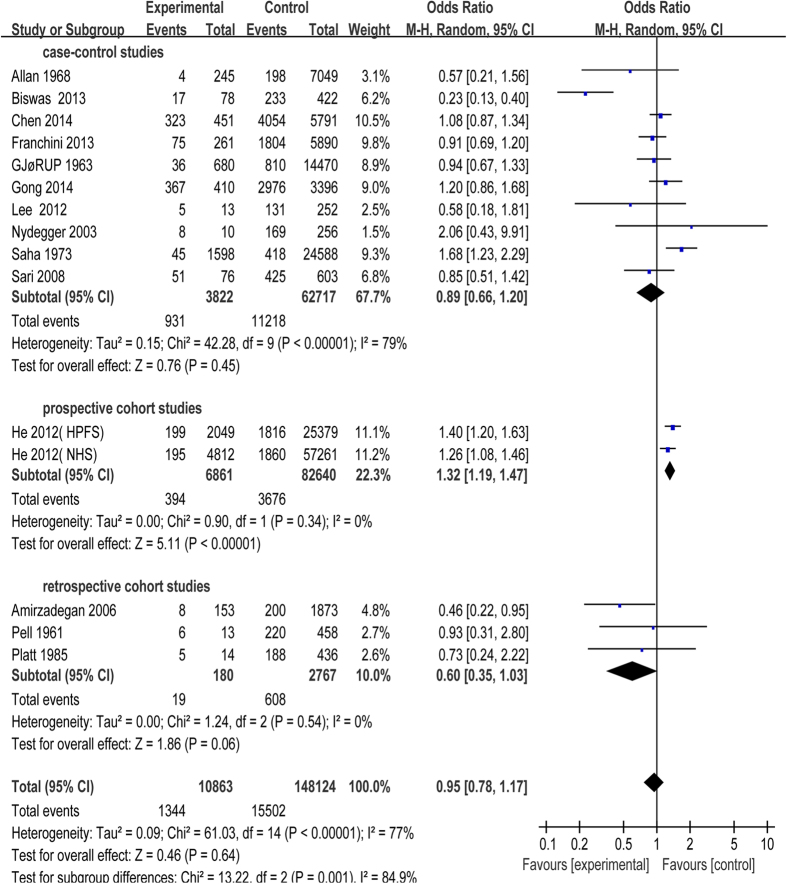
Forest plot of blood group AB.

**Figure 5:**
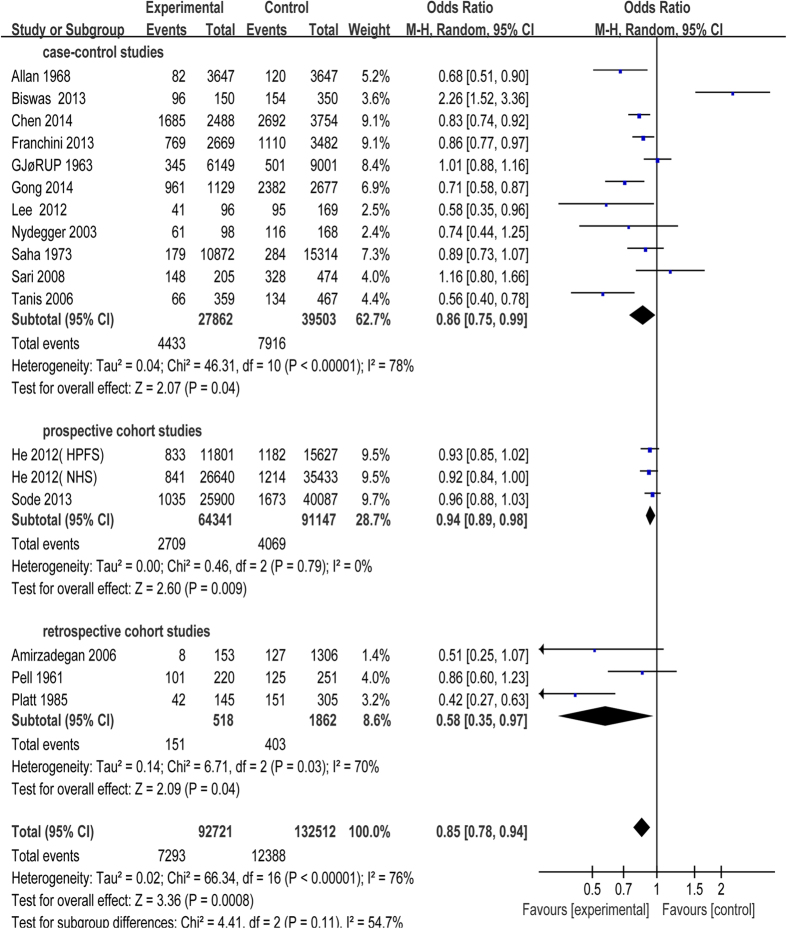
Forest plot of blood group O.

All the 17 studies were included in this meta-analysis. Because of unnegligible heterogeneity in them, we conducted a subgroup analysis according to the research types (case-control study, prospective or retrospective cohort study) and used random-effect model^27^. Risk of CAD was significantly increased in patients with blood group A compared with blood group non-A (odds ratio (OR) = 1.14, 95% confidence intervals (CI) = 1.03 to 1.26, p = 0.01). Subjects in blood group A had a statistical increase in CAD incidence in case-control studies (OR = 1.14, 95% CI = 1.04 to 1.26, p = 0.005) with moderate heterogeneity (I^2^ = 45%), while there was no statistical difference between blood group A and non-A in cohort studies (Fig. 2). Besides, risk of CAD had no statistical significant difference in patients with blood group B, AB compared with non-B, non-AB, respectively (Figs 3 and 4). Whereas, in contrast to the result of blood group A, blood group O was proved to be a protective factor in our analysis, presenting a decrease of CAD risk (OR = 0.85, 95% CI = 0.78 to 0.94, p = 0.0008). Our analysis found that there was statistical significant difference in CAD incidence in case-control studies (OR = 0.86, 95% CI = 0.75 to 0.99, p = 0.04) in spite of high heterogeneity (I^2^ = 78%), which is similar to prospective cohort studies (OR = 0.94, 95% CI = 0.89 to 0.98, p = 0.009) with no heterogeneity (I^2^ = 0). However, there was no statistical significant difference in CAD incidence in retrospective cohort studies (OR = 0.58, 95% CI = 0.35 to 0.97, p = 0.04) with high heterogeneity (I^2^ = 70%) (Fig. 5). In the sensitivity analysis, exclusion of any single study did not substantively alter the overall result in blood group A, B, AB and O. In order to exclude the effect of established positive relationship between ABO blood group and MI, we removed the studies^12^,^16^,^17^,^19^,^20^,^21^,^22^,^23^,^24^ which only paid attention to MI patients and found the similar relationship between ABO blood group and CAD as before, namely, A (OR = 1.05, 95% CI = 1.00 to 1.10, p = 0.03) and O (OR = 0.89, 95% CI = 0.85 to 0.93, p < 0.00001).

Furthermore, risk of MI was significantly higher in blood group A (OR = 1.24, 95% CI = 0.97 to 1.59, p = 0.08) compared with non-A group. Nevertheless, patients with blood group B or AB compared to non-B or non-AB, respectively, had no statistical differences in MI incidence (OR = 0.94, 95% CI = 0.74 to 1.18, p = 0.59; OR = 1.11, 95% CI = 0.91 to 1.35, P = 0.31). However, an overall effect was detected to be statistically different when comparing blood group O with non-O for the risk of MI (OR = 0.81, 95% CI = 0.69 to 0.94, p = 0.007).

### Publication bias

**Figure 6:**
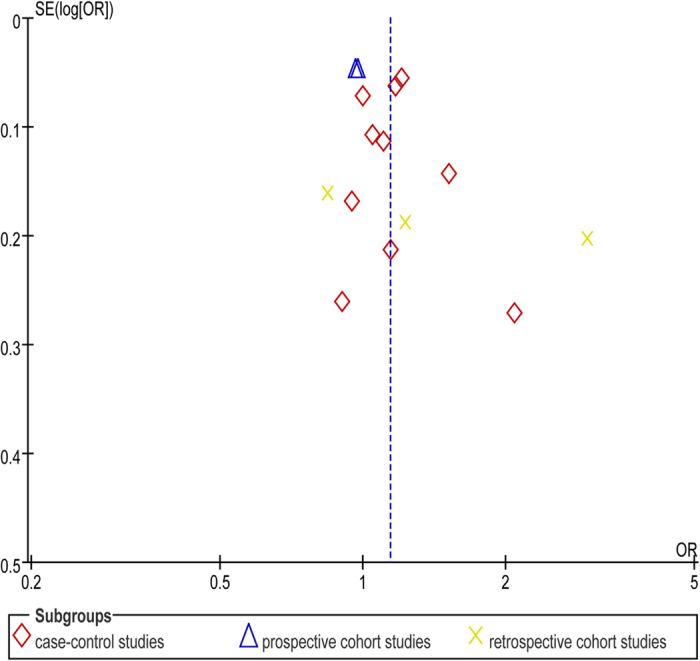
Funnel plot of blood group O.

We generated a funnel plot to assess publication bias. Exploration for the funnel plot of the blood group O in CAD suggested no asymmetry. No obvious evidence of publication bias was present in the comparison of blood group O (Fig. 6).

## Discussion

Previous systematic reviews and meta-analysis paid more attention to the relationship between MI and ABO blood group, but the link of ABO blood group system to CAD was rarely evaluated. Besides, almost all available studies principally focused on blood type non-O and O. Hence, the relation between ABO blood group and risk of CAD is worthy to be assessed scientifically and strictly.

Our meta-analysis involved 16 articles (17 studies) covering 225,810 individuals. It was suggested that the risk of CAD in blood group A was mildly increased compared with that in blood group non-A (OR = 1.14). Meanwhile, we investigated the relationship of blood group B, AB compared with non-B, non-AB, respectively, but failed to confirm statistical difference. Moreover, our results indicated that the risk of CAD in blood group O was significantly lower than that in non-O groups (OR = 0.85), which is similar to previous studies^8^.

To our knowledge, this is the first meta-analysis involved the relationship between the risk of CAD and blood group A and non-A. Several clinical studies have provided direct evidence with different results. Whincup *et al*.^28^ found that the incidence of ischaemic CAD was higher in those with blood group A than that with blood group non-A (OR = 1.21, 95% CI = 1.01 to 1.46). A study from Wazirali *et al*.^29^ suggested that blood group A was associated with a substantially increased risk of CAD, which is independent of conventional cardiovascular risk factors. Whereas, another research did not support this association and indicated that the risk of CAD in blood group A was lower than that in other blood groups^30^. As we known, meta-analyses provide advance over traditional single studies. That is a reason why we performed a meta-analysis for further evaluating the relation of blood group A to the risk of CAD. In our study, we affirmed blood group A was a risk factor, which is more convincing and reliable. Similar evidence was more robust in the analysis for MI incidence (OR = 1.24).

Our study showed a significantly reduced risk of CAD in individuals with blood group O compared with that with blood group non-O (OR = 0.85, 95% CI = 0.78 to 0.94, p = 0.0008). Evidence was more obvious when we performed an analysis concerning the relationship between ABO blood group and MI (OR = 0.81, 95% CI = 0.69 to 0.94, p = 0.007). In fact, non-O blood group as an independent risk factor was already confirmed in other systematic reviews, too^8^,^9^,^10^. Wu *et al*.^9^ performed a meta-analysis with regard to the relation of ABO blood group to MI and angina in 2008. In their study, taking group O as index, group A and non-O were related to an increase in MI risk (OR = 1.29, 95% CI = 1.16 to 1.45, p < 0.00001, OR = 1.25, 95% CI = 1.14 to 1.36, p < 0.00001), while no similar effect was found in the risk of angina. Furthermore, a meta-analysis by Dentali *et al*.^8^ found that patients with blood group non-O presented a higher prevalence of MI than that with blood group O (OR = 1.28, 95% CI = 1.17 to 1.40, p < 0.001). Takagi *et al*.^10^ enrolled 10 studies with a total of 174,945 participants and demonstrated a 14% increase in CAD incidence in individuals with blood group non-O compared to that in blood group O (OR = 1.14, 95% CI = 1.04 to 1.25, p < 0.006). All in all, the quantitative results from these meta-analyses and our one provided plenty of evidence on the close relationship between risk of CAD and blood group non-O.

The underlying mechanism of the relationship between blood group O and CAD has been clarified. ABO antigen may affect plasma levels of vWF and coagulation factor VIII^7^, and blood group non-O has the lowest expression of O antigen and relatively higher levels of vWF and factor VIII^31^. That blood group O is a potentially important genetic risk factor for bleeding^32^, which also supports this mechanism theory. Another biologically plausible mechanism involves in glycotransferase-deficient enzyme which renders the ABO blood group to encode O phenotype, resulting in protection of subjects from MI risk^33^. The latest study reveals that serum lipid mediates the effect of ABO blood group on CAD. In fact, blood group A is one of the risk factors of CAD mainly due to higher serum total cholesterol (TC) concentration in subjects^28^. Our recent study also indicated that there is an association between blood group A and risk of CAD, and around 10.5% of the effect of blood group A on CAD is mediated by TC levels^34^.

It was mentioned that there were several potential limitations in this study. Firstly, there was certain heterogeneity between various studies. Although we performed subgroup analyses, it was still different among the studies in blood testing methods and diagnostic criteria of CAD, race, life and eating habits, religious beliefs, socio-economic patterns, and concern of the disease, which might result in the heterogeneity. Secondly, we did not find unpublished studies, which may bring about publication bias.

In conclusion, this updated meta-analysis suggests that blood group A and non-O are associated with an increased risk of CAD. However, considering the heterogeneity of included studies and limited number of studies, more rigorous studies with high quality are needed to give high level of evidence to confirm this association.

## Methods

This meta-analysis was performed according to the MOOSE group guidelines of observational meta-analyses^35^.

### Data sources and searches

Two reviewers (Zhuo Chen and Sheng-Hua Yang) searched Pubmed and the Cochrane Library from their inception to August 15, 2015 in order to identify all existing literature which assessed the association between ABO blood group and CAD. Mesh vocabulary and free text terms were used for each database with relevant key words such as blood grouping and cross-matching, ABO blood group system, blood group antigens, myocardial ischemia, myocardial infarction, acute coronary syndrome and angina pectoris. Language was limited to English. There was no limitation of country and publication date.

To ensure comprehensive acquisition of studies, the reference lists of the included articles were also manually screened to identify additional eligible studies. Manual searches were also performed on other databases, including Web of Science, and Google Scholar. Furthermore, databases of ongoing trials were also searched: Clinical Trials.gov (http://clinicaltrials.gov/) and Current Controlled Trials (http://www.controlled-trials.com/).

### Study selection

Studies were independently identified by two reviewers (Zhuo Chen and Sheng-Hua Yang) according to inclusion criteria. Disagreements were resolved through discussion and decided by a third reviewer. Both case-control and cohort studies were included if they met all the following criteria: 1) patients with CAD or even MI; 2) separate data for patients with or without CAD were provided; 3) diseases were objectively diagnosed in line with the diagnosis level at the time; 4) a clear extractable ABO blood group typing. Patients included were regardless of age and race.

### Data extraction and quality assessment

The retrieved papers were subjected to a rigorous extraction by two authors (Zhuo Chen and Sheng-Hua Yang) independently according to a predesigned form. Disagreements were resolved by consensus or consulted from the third author (Hao Xu). We did not try to contact authors to obtain unpublished data. The methodological quality of studies was assessed using the NOS checklist for observational studies^36^. We rated cohort studies a maximum of 4 stars for selection, 2 stars for comparability, and 3 stars for outcome assessment. The maximum score of case-control studies for selection, comparability, and exposure assessment was 4, 2, 3, respectively, too. The highest score is 9, and more stars meant better quality.

### Data analysis and synthesis

Revman 5.2 software (The Cochrane Collaboration, Oxford, UK) was used for data analyses. We presented dichotomous data as OR and its 95% CI. Data were assessed by both random and fixed effect models, but only the random effect analyses were reported if the heterogeneity was significant evaluated by the I^2^ statistic which assessed the appropriateness of pooling all studies^27^. A funnel plot was used to assess publication bias.

## Additional Information

**How to cite this article**: Chen, Z. *et al*. ABO blood group system and the coronary artery disease: an updated systematic review and meta-analysis. *Sci. Rep*. **6**, 23250; doi: 10.1038/srep23250 (2016).

## Figures and Tables

**Table 1:** Characteristics of included studies.

Study year, reference	Type of study	Total number of subjects	Age	Gender	Race	Patients with events	Controls
Chen 2014	case-control study	6242	64.11 ± 11.42	71.4% are men, 28.6% are women	Chinese	consecutive patients undergoing diagnostic or interventional coronary angiography, who were finally diagnosed CAD or MI	consecutive patients undergoing diagnostic or interventional coronary angiography, who were not diagnosed CAD or MI
Gong 2014	case-control study	3806	53.9 ± 9.9	male (71%)	Chinese	angiographically documented CAD or diagnosed as MI	the patients without CAD or MI
Biswas 2013	case-control study	500	cases (mean: 54.71 years) and controls (mean: 54.49 years) (*P* > 0.05)	females constituted 18.4% of the cases and 16.8% of the controls and males constituted 81.6% of cases and 83.2% of the controls (*P* > 0.05)	Indian Bengali adults	Patients having typical angina and evidence of ischemia or infarction after electrocardiographic study, tread mill test, stress echo and echocardiographic study	controls comprised the spouses, neighbors, and people from same work place of the patients, with the same sociocultural background, in whom the clinical history, the objective search for signals of CHD, and the electrocardiographic as well as echocardiographic examinations did not suggest the presence of that disease
Sode 2013	two prospective cohort studies	the Copenhagen general population study(25900) + the Copenhagen city heart study (40097) = 65,997	aged 20–100 years	women (36,562); men (29,439)	white and of Danish descent	the patients with myocardial infarction (defined as ICD-8 code 410 and ICD-10 codes I21–I22)	the general population of Copenhagen without diagnoses of MI and IHD
Franchini 2013	case-control study	6151	mean age: 77 years in the CHD group; 34 years in the control group	females: 34.9% in the CHD group; 46.7% in the control group	Italians	the patients with coronary heart disease (CHD)	the healthy general population
He 2012	two prospective cohort studies	[NHS]62073	aged 30–55 years	women	American (different ethnicities)	incident cases of CAD	patients in the same cohort who did not occur coronary heart disease
		[HPFS]27428	aged 40 to 75 years	male	American (different ethnicities)	incident cases of CAD	patients in the same cohort who did not occur coronary heart disease
Lee 2012	case-control study	265	men younger than 45 years and women younger than 55 years	52.1% are men, 47.9% are women	Chinese	subjects who underwent coronary angiography with documented CAD	subjects without angiographically demonstrable lesions served as controls
Sari 2008	case-control study	679	mean age 56.7 ± 11.7 in case, mean age 58.1 ± 12.2 in control	80.3% men in patients, 82.7% men in control	Turkish	patients with acute ST elevation MI	subjects without known CAD
Tanis 2006	case-control study	826	between 18 and 49 yr of age	women	Netherlands	the patients suffered from MI	women contacted by random-digit dialing, stratified for age, index year for MI, and area of residence
Amirzadegan 2006	retrospective cohort study	2026	a mean age of 59 years	1512 males (75.4%) and 494 females (24.6%)	Iranian	the patients with premature CAD defined as development of CAD under 45 years old	the patients without premature CAD defined as development of CAD under 45 years old
Nydegger 2003	case-control study	266	median age 57.0 years; range 32–72 years	87.6% men in patients, 88.8% men in controls	Caucasian	survivors of an acute myocardial infarction that had occurred at least 2 months before inclusion in the study	healthy Caucasian without a history of thromboembolic events or tendency to bleed, were frequency-matched to the cases by age (SD 5 years) and sex
Platt 1985	retrospective cohort study	450	66/450 (age < 65 yr) 384/450 (age >= 65 years)	139/450 (male)	German	the patients with cardiac infarction	sample of the German population
Saha 1973	case-control study	26186	age >= 20 years	NR	Chinese Malays Indians	the patients with myocardial Infarction	the healthy individuals (matched for race and sex)
Allan 1968	case-control study	7294	NR	the ABO blood group distribution is almost exactly the same for men and women.	British	the patients with myocardial infarction	consecutively-registered blood donors
Gjørup 1963	case-control study	15,150	NR	610/846 (Male) 236/846 (women) in case group	Danes	the patients with coronary occlusion	blood donors from the same area
Pell 1961	retrospective cohort study	471	from 17 through 64 years	NR	American	the medical records of the 438 employees (coronary patients) who had a first coronary attack of coronary thrombosis and or myocardial infarction during 1957 and 1958	the records of the 438 matched controls (the controls were drawn at random from a complete listing of company employees with the aid of a table of random numbers, and were matched to each case in our series by age, sex, payroll classification, and geographical location) were reviewed to compare the occurrence of certain chronic diseases in the two groups

NR: not report.

CAD: coronary artery disease.

MI: myocardial infarction.

**Table 2:** Blood types distribution and outcome definitions of included studies.

Study year, reference	A, non-A	B, non-B	AB, non-AB	O, non-O	Outcome definitions
Chen 2014	1227/1692, 3150/4550	1142/1611, 3235/4631	323/451, 4054/5791	1685/2488, 2692/3754	CAD: Significant CAD indicated by >50% stenosis in ≥1 coronary artery in angiography.
Gong 2014	909/1026, 2434/2780	1106/1241, 2237/2565	367/410, 2976/3396	961/1129, 2382/2677	significant angiographically documented CAD as having >50% diameters stenosis in ≥1 major coronary artery
Biswas 2013	60/114, 190/386	77/158, 173/342	17/78, 233/422	96/150, 154/350	CAD: typical angina and evidence of ischemia or infarction
Sode 2013				1035/25900, 1673/40087	myocardial infarction was defined as ICD-8 code 410 and ICD-10 codes I21-I22.
Franchini 2013	856/2604, 1023/3547	179/617, 1700/5534	75/261, 1804/5890	769/2669, 1110/3482	coronary heart disease (CHD)
He 2012 [NHS]	723/22358, 1332/39715	296/8263, 1759/53810	195/4812, 1860/57261	841/26640, 1214/35433	Incident cases of CAD (non-fatal MI or fatal CHD): A physician unaware of the self-reported risk factor status verified the report of MI through review of medical/hospital records by using the World Health Organization criteria of symptoms and either typical ECG changes or elevated cardiac enzymes. 26 Fatal CAD was confirmed by medical records or autopsy reports, or by CAD listed as the cause of death on the death certificate and there was evidence of previous CHD in the records.
He 2012 [HPFS]	737/10213, 1272/17215	246/3365, 1769/24063	199/2049, 1816/25379	833/11801, 1182/15627	
Lee 2012	54/85, 82/180	36/71, 100/194	5/13, 131/252	41/96, 95/169	presence of CAD was defined as >50% stenosis in at least 1 major coronary branch, on coronary angiography.
Sari 2008	205/295, 271/384	72/103, 404/576	51/76, 425/603	148/205, 328/474	MI: based on typical chest pain for at least 30 min, ST elevation of 0.2 mV or more in at least two contiguous electrocardiogram leads and confirmatory elevations of at least two-fold in serum creatine kinase-MB isoenzyme levels
Tanis 2006				66/359, 134/467	acute MI
Amirzadegan 2006	60/650, 148/1376	59/503, 149/1523	8/153, 200/1873	8/153, 127/1306	premature CAD defined as development of CAD under 45 years old
Nydegger 2003	87/133, 90/133	21/25, 156/241	8/10, 169/256	61/98, 116/168	acute myocardial infarction
Platt 1985	137/253, 56/197	9/38, 184/412	5/14, 188/436	42/145, 151/305	cardiac infarction: NR
Saha 1973	119/6506, 344/19680	120/7210, 343/18976	45/1598, 418/24588	179/10872, 284/15314	myocardial Infarction: all cases of myocardial infarction as confirmed by clinical, electrocardiographic, and biochemical investigations
Allan 1968	92/2607, 110/4687	774/795, 178/6499	4/245, 198/7049	82/3647, 120/3647	myocardial Infarction: unequivocal electrocardiographic evidence of recent infarction, or if appropriate rises in serum transaminase levels occurred where myocardial changes were masked, as, for example, by a bundle-branch-block pattern
Gjørup 1963	372/6671, 474/8479	93/1650, 753/13500	36/680, 810/14470	345/6149, 501/9001	coronary occlusion: typical ECG abnormalities combined with characteristic pains
Pell 1961	98/192, 128/279	21/46, 205/425	6/13, 220/458	101/220, 125/251	MI:NR

NR: not report.

CAD: coronary artery disease.

MI: myocardial infarction.

**Table 3:** Newcastle-Ottawa Scale table.

Study reference	Selection	Comparability	Measurement	Total
Case-control studies				
Chen 2014	4	1	2	7
Gong 2014	2	1	3	6
Biswas 2013	4	1	3	8
Franchini M 2013	2	1	2	5
Lee 2012	4	1	2	7
Sari 2008	3	2	3	8
Tanis 2006	2	1	2	5
Nydegger 2003	1	0	1	2
Platt 1985	2	0	1	3
Jick 1978	3	1	2	6
SAHA 1973	2	1	2	5
ALLAN 1968	3	1	2	6
Gjørup 1963	3	1	1	5
Pell 1961	2	1	0	3
cohort studies				
Sode 2013	3	2	2	7
He 2012 [NHS]	3	2	3	8
He 2012 [HPFS]	3	2	3	8
